# A Bayesian Network approach to study the relationships between several neuromuscular performance measures and dynamic postural control in futsal players

**DOI:** 10.1371/journal.pone.0220065

**Published:** 2019-07-25

**Authors:** Iñaki Ruiz-Pérez, Francisco Ayala, José Miguel Puerta, Jose L. L. Elvira, Mark De Ste Croix, Sergio Hernández-Sánchez, Francisco Jose Vera-Garcia

**Affiliations:** 1 Department of Sport Sciences, Sports Research Centre, Miguel Hernandez University of Elche, Elche, Spain; 2 Postdoctoral fellow from Seneca Foundation, Murcia, Spain; 3 Department of Computer Systems, University of Castilla-La Mancha, Albacete, Spain; 4 School of Sport and Exercise, University of Gloucestershire, Gloucester, United Kingdom; 5 Department of Pathology and Surgery, Physiotherapy Area, Miguel Hernandez University of Elche, Alicante, Spain; The Wingate College of Physical Education and Sports Sciences at the Wingate Institute, IL, ISRAEL

## Abstract

**Purpose:**

The purpose of this study was to analyse the relationship between several parameters of neuromuscular performance with dynamic postural control using a Bayesian Network Classifiers (BN) based analysis.

**Methods:**

The y-balance test (measure of dynamic postural control), isokinetic (concentric and eccentric) knee flexion and extension strength, isometric hip abduction and adduction strength, lower extremity joint range of motion (ROM) and core stability were assessed in 44 elite male futsal players. A feature selection process was carried out before building a BN (using the Tabu search algorithm) for each leg. The BN models built were used to make belief updating processes to study the individual and concurrent contributions of the selected parameters of neuromuscular performance on dynamic postural control.

**Results:**

The BNs generated using the selected features by the algorithms correlation attribute evaluator and chi squared reported the highest evaluation criteria (area under the receiver operating characteristic curve [AUC]) for the dominant (AUC = 0.899) and non-dominant (AUC = 0.879) legs, respectively.

**Conclusions:**

The BNs demonstrated that performance achieved in the y-balance test appears to be widely influenced by hip and knee flexion and ankle dorsiflexion ROM measures in the sagittal plane, as well as by measures of static but mainly dynamic core stability in the frontal plane. Therefore, training interventions aimed at improving or maintaining dynamic postural control in elite male futsal players should include, among other things, exercises that produce ROM scores equal or higher than 127° of hip flexion, 132.5° of knee flexion as well as 34° and 30.5° of ankle dorsiflexion with the knee flexed and extended, respectively. Likewise, these training interventions should also include exercises to maintain or improve both the static and dynamic (medial-lateral plane) core stability so that futsal players can achieve medial radial error values lower than 6.69 and 8.79 mm, respectively.

## Introduction

The y-balance is a reliable [1,2], time efficient and portable (field-based) test widely used to assess dynamic postural control [3]. This test is usually included as part of an injury risk battery in both clinical and sporting contexts, primarily based on the fact that several studies [4–8], although not all [9,10], have reported that poor performance and bilateral asymmetries may be considered as valid predictors for identifying athletes at high risk of non-contact lower extremity injuries (mainly knee and ankle injuries). Thus, Butler et al. [5] found that collegiate football players were 3.5 times more likely to suffer a non-contact lower extremity injury when they reported y-balance normalized composite scores below 89.6%. Similarly, Calvo-Gonel et al. [6] reported that elite football players with bilateral asymmetries equal to or greater than 4 cm in the posteromedial direction of the y-balance test had a 3.86 greater probability of suffering a non-contact injury than those who did not. Furthermore, the y-balance test is sensitive enough to differentiate between different levels of competition [11–13] and sporting populations [14]. Elite football players have demonstrated better y-balance scores than their non-elite peers [11,12] and when compared with other sporting populations, footballers have performed better on either leg [14].

The y-balance test involves maintaining single-legged balance whilst simultaneously reaching as far as possible with the contralateral leg in three directions (anterior, posterolateral and posteromedial). Potentially, the execution of this test might require, among others, adequate levels of hip and knee strength, power, trunk or core stability, coordination and lower extremity ranges of motion (ROM). With the aim of improving the design of training interventions, some studies have explored the individual contribution of certain measures of knee strength [15–17], hip strength [17–19], lower extremity power [20], core stability [17] and lower extremity ROMs [17,21] on y-balance test performance using linear regression models in different cohorts of athletes. However, these studies have reported conflicting results that might not permit clinicians, physiotherapists and physical trainers to make general training recommendations. For example, Booysen, Gradidge & Watson [15] did not show any relationship between the isokinetic strength of the knee flexors and extensors and the y-balance test score in professional football players, whereas Lockie et al. [16] did find a positive and statistically significant correlation (r = 0.50; p = 0.008) between the isokinetic strength of the knee extensors and the y-balance test performance in amateur team sport athletes. The conflicting results might be partially attributed to the different sport modalities and levels of competition (i.e. amateur vs. professional or elite) of the athletes recruited in each study. In particular, the differences in technical skills, specific movements, training load and physical capacities among sports and levels of competition may predispose participants to individual chronic musculoskeletal adaptations, thus influencing some neuromuscular measures and their subsequent impact on the y-balance test performance. Therefore, it may be necessary a sport-specific and level of competition-based analysis of which neuromuscular parameters contribute to y-balance test performance in order to design effective dynamic postural control training interventions.

Despite being one of the most popular sports worldwide [22,23] and being ranked among the top ten non-contact lower extremity injury-prone sports [24], an analysis of the influence of the main modifiable measures of neuromuscular performance (i.e. hip and knee strength, core stability, lower extremity ROMs) on y-balance test scores in futsal players has not been undertaken. In terms of sport performance, futsal players might be a target group for dynamic postural control training programmes since they are required to perform repetitively high intensity unilateral movements such as sudden acceleration and deceleration tasks, rapid changes of direction, kicking and tackling [25,26].

The existing literature has predominantly used traditional lineal regression analyses to explore statistical associations and to our knowledge no studies have used contemporary statistical techniques, such as Bayesian Networks Classifiers (BNs) (also referred to as causal networks or belief networks) to provide evidence of relationships of dependency and conditional independence between different measures or variables [27]. In contrast to traditional statistics, BNs not only provide statistical models describing the relationships between variables from empirical data (as a way of representing uncertainty), but construct graphical probabilistic models (directed acyclic graphs) based on the underlying structure in which variables are represented by nodes and their relationships of dependency are symbolized by arrows or arcs [28]. Thus, the graphical representation of BNs captures the compositional structure of the relations and the general aspects of all probability distributions that factorize according to that structure [29]. Furthermore, BNs allow making inference or relevance analysis/reasoning in a natural manner and within a dynamic context to generate intercausal reasoning, that is to say, adding new evidence to the model in order to study the impact of the new relationships generated in the class variable. Therefore, the use of a BN based analysis to study the relationships of dependency and conditional independence between the main modifiable measures of neuromuscular performance and dynamic postural control and particularly the subsequent graph generated will help clinicians, physiotherapists and physical trainers to understand this complex phenomenon better. In addition, the BN model built could be used to make belief updating processes (by adding new evidence [the scores obtained by an athlete in the different neuromuscular performance tests]) in order to study the concurrent and individual contribution of the neuromuscular factors on the dynamic postural control of each futsal player and thus allowing the design of individualised training programs.

Therefore, the main purpose of the current study was to analyse the relationships between several parameters of neuromuscular performance with dynamic postural control (measured through the y-balance test) using a BN based analysis in a cohort of elite futsal players.

## Method

### Participants

A total of 44 elite male futsal players from four different teams (16 players from a club engaged in the First [top] National Spanish Futsal division and 28 players from three clubs engaged in the Second National Futsal division) completed this cross-sectional study (convenience sampling). To be included, all participants had to be free of pain at the time of the study and currently involved in futsal-related activities. Participants were excluded if they reported the presence of any lower extremity injury within the last month, a current upper respiratory tract infection, any bone or joint abnormalities, any uncorrected visual and vestibular problems and/or a concussion within the last three months [15]. The study was conducted at the end of the pre-season phase in 2015 and 2016 (September). Before any participation, experimental procedures and potential risks were fully explained to the participants in verbal and written form, and written informed consent was obtained from participants. An Institutional Research Ethics committee approved the study protocol prior to data collection (DPS.FAR.01.14), conforming to the recommendations of the Declaration of Helsinki.

### Testing procedure

Prior to the neuromuscular testing, all participants performed a standardised dynamic warm-up designed by Taylor et al. [30]. Three to 5 min after the dynamic warm-up was carried out, participants completed five different neuromuscular assessments in the following order: 1) dynamic postural control; 2) isometric hip abduction and adduction strength; 3) lower extremity joint ROMs; 4) core stability; and 5) isokinetic knee flexion and extension strength.

Dynamic postural control was measured using the y-balance test (Y-Balance Test, Move2Perform, Evansville, IN) (composite score) and followed the guidelines proposed by Shaffer et al. [2]. After having completed a 2 min practise of the testing procedure, players were allowed a maximum of five trials to obtain three successful trials for each reach direction (anterior, posteromedial and posterolateral). To obtain a global measure of the dynamic postural control performance, the greatest distance reached in each direction was normalised (by dividing by leg length) and then averaged (by multiplying by 100) to establish a composite balance score.

Isometric hip abduction and adduction peak torque of the dominant and non-dominant leg were assessed using a portable handheld dynamometer (Nicholas Manual Muscle Tester, Lafayette Indiana Instruments) with the participant lying in a supine position on a plinth with legs extended, following the methods described by Thorborg et al. [31]. Participants performed two practice trials (50 and 80% of the self-perceived isometric maximal voluntary contraction) and then three 5s isometric maximal voluntary contraction trials for each hip movement. The best trial was used for the subsequent statistical analyses.

Likewise, passive hip flexion with knee flexed and extended, extension, abduction, external and internal rotation; knee flexion; and ankle dorsiflexion with knee flexed and extended ROMs of the dominant and non-dominant leg were assessed following the methods previously described [32]. The best score for each test was used in the subsequent analyses.

An unstable sitting protocol was used to assess participant’s core stability, determined as the ability to control trunk posture and motion while sitting, following the methods previously described by Barbado et al. [33]. Briefly, after a familiarization period (2 min), participants performed different static and dynamic tasks while sitting on an unstable seat. All tasks were performed twice. The duration of each trial was 70s and the rest period between trials was 1 min. The mean radial error was used as a global measure to quantify the trunk/core performance during the trials.

Finally, isokinetic concentric and eccentric torques during knee extension and flexion actions in both legs were determined (Biodex System-4, Biodex Corp., Shirley, NY, USA) following the methods employed by Ayala et al. [34]. In each of the three trials at each velocity (60°/s and 180°/s for concentric muscle actions and 30°/ and 60°/s for eccentric muscle actions), the peak torque was reported as the single highest torque value achieved. For each peak torque variable, the best of the three trials at each velocity was used for subsequent statistical analysis. When a variation >5% was found in the peak torque values between the three trials, the mean of the two most closely related torque values was used for the subsequent statistical analyses.

Appendix 1 summarizes the list of variables recorded from each assessment procedure (and it also shows the abbreviations that have been used within the manuscript). Each of the 6 testers who took part in this study conducted the same tests throughout all the testing sessions. All testers had more than 4 years of experience in using the neuromuscular assessments.

### Statistical analysis

Prior to building the BN of each leg, all variables were discretized as this has been shown to be an effective measure to improve the performance of several BN and logistic regression techniques [35]. Thus, both class variables (y-balance composite score of the dominant and non-dominant legs) were discretized into two intervals (high risk and low risk of injury) according to the cut-off score of 89.6% reported by Butler et al. [5], in which composite scores below 89.6% indicate that players are 3.5 times more likely to suffer a non-contact lower extremity injury (100% of sensitivity and 71.7% of specificity). A statistician experienced in running BN analysis carried out the discretization of the continuous variables using a visual inspection of their histogram (in which each instance was colored [blue or red] according to their relationship to each interval of the class variable [high risk or low risk]) which allowed identification of a clear cut-off point. Thus for the y-balance composite score of the dominant and non-dominant leg, six and eight variables were discretized into two intervals, respectively. For those variables in which a clear cut-off score was not visually identified, the unsupervised discretization algorithm available in the WEKA Data Mining software was applied using the equal frequency binning approach (three cut point intervals). Three intervals were selected in order to reflect taxonomy of low, moderate and high scores that might make the final models more comprehensible. Appendix 1 shows a description of all variables recorded to build the BNs.

In order to build the BN of each leg that allows the classification of futsal players into one of the two injury risk categories (low risk or moderate risk) previously defined according to their dynamic postural control scores, we used the well-known WEKA (Waikato Environment for Knowledge Analysis) Data Mining software. To build the BN the score + search approach was used [36]. Specifically, the Tabu search algorithm as a search engine [37] coupled with the BDeu score [38] was selected to build the structure of both BNs (dominant and non-dominant leg). This algorithm explores the search space starting from a network structure and adding, deleting, or reversing one arc at a time until the score can no longer be improved. Thus, the Tabu search algorithm is a modified hill climbing algorithm able to escape local optima by selecting a network that minimally decreases the score function. Neither expert knowledge nor prior knowledge of the system under study was taken into account in the model selection process in order to prevent the model from encoding the prior information instead of the information in the data. As the Tabu search is a stochastic algorithm, the final model was obtained by repeating the structure learning several times (in our case 1,000 times). A large number of network structures were explored (1,000 BNs) to reduce the impact of locally optimal (but globally suboptimal) network learning. The networks learned were averaged to obtain a more robust model. A conditional probability distribution was obtained for each node.

The performance of the BNs was assessed using a 5-fold stratified cross validation technique. That is, we split the dataset into 5 folds, each one containing 20% of the patterns of the dataset. For each fold, the BN was trained with the examples contained in the remaining folds and then tested with the current fold. A wide range of performance measures can be obtained from the stratified cross validation technique. A well-known approach to unify these measures and to produce an evaluation criterion is to use the area under the Receiver Operating Characteristic Curve (AUC). In particular, the AUC corresponds to the probability of identifying which one of the two stimuli is noise and which one is signal plus noise correctly [39]. Thus, the AUC was used as a single measure of BNs´ performance.

However, and before learning the BNs, a feature selection process was carried out to reduce the dimensionality of the feature space and eliminate irrelevant, weakly relevant and/or redundant features. In other words, the aim of this pre-learning process was to find the minimal subset of attributes such that the resulting probability distribution of data classes is close to the original distribution obtained using all attributes and that they do not decrease the accuracy of the model significantly [40]. Feature selection algorithms are separated into three categories: a) *the filters* which extract features from the data without any learning involved, b) *the wrappers* that use learning techniques to evaluate which features are useful, and c) *the embedded techniques* which combine the feature selection step and the classifier construction [41,42]. A priori it is not possible to determine with certainty which category of the feature selection algorithms might be applied to address each problem more accurately. Thus, it has been suggested that an appropriate approach may be to analyze and compare the accuracy of the models built for a given classifier (in our case the Tabu search algorithm) to which different feature selection techniques have been previously applied and then select the best performing BN-based feature selection method [43–45]. Accordingly, the behavior of numerous feature selection algorithms coming from the filter and wrapper categories were analyzed and compared (using the metaclassifier “attribute selected classifier” available in Weka´s repository) in order to select the best performing BN to describe the relationships between the main measures of neuromuscular performance and dynamic postural control. For those filter algorithms in which a ranker search technique is required (e.g. chi squared attribute evaluator and correlation attribute evaluator techniques), it was set up to select the top-10 ranked features so that a comprehensible and straightforward model could be developed. Once the top-10 ranked features were determined, the performance of these filter algorithms were assessed by using the top-10, 9, 8, 7 … and 2 features and then compared in order to find the minimal subset of features with the best performance. On the other hand, the search algorithms used for the wrapper algorithms were the Best First (backward direction) and Greedy Stepwise (backward direction) and as base classifier the following three classifier algorithms were selected: Naïve Bayes, C4.5 and Support Vector Machine. The accuracy scores of all the possible combinations for the wrapper algorithms were compared and the best performing model was finally selected.

The BNs were implemented using SAMIAM (Sensitivity Analysis Modeling Inference and More) software (2013) to obtain a graphical interface for manipulating the probabilistic network.

Once the BNs were built, different configurations of variable's values where entered with the aim of studying different intercausal (interactions among different causes of the same effect) and causal (predictions from causes to effects) reasoning scenarios.

## Results

Tables 1 and 2 show the accuracy scores obtained by the 11 feature selection algorithms used to build different dynamic postural control BNs (y-balance test composite score) for the dominant and non-dominant leg, respectively. For the dynamic postural control of the dominant leg, the feature selection algorithm “correlation attribute evaluator” (which evaluates the worth of an attribute by measuring the correlation [Pearson's] between it and the class) belonging to the *filters* category was the algorithm that built the BN with the highest accuracy score (AUC = 0.899). The dynamic postural control BN built for the non-dominant leg after the application (pre-processing) of the “chi squared” feature selection algorithm (that evaluates the worth of an attribute by computing the value of the chi-squared statistic with respect to the class), also belonged to the filters category, and had the highest AUC scores (0.879). Furthermore, these two feature selection algorithms used six and ten variables to build the dynamic postural control BNs that showed the highest performance for the dominant and non-dominant leg, respectively.

**Table 1:** Comparisons among the accuracy scores obtained by all the BN-based feature selection methods for the dominant leg. In grey is highlighted the best performing BN.

Feature selection algorithm	Search technique	AUC	N° of features selected	Description in ascending (from more to less important/relevant) order
-	-	0.865	31	S1 Table
Correlation-based feature subset evaluator	Best First	0.858	5	ISOK-PT-ECC-KF_180_, CS-NF, CS-ML, ROM-HF_KF_ and ROM-KF
Chi squared attribute evaluator	Ranker	0.835	4	ROM-KF, ROM-HF_KF_, CS-ML and ROM-HE
Classifier attribute evaluator (Naïve Bayes)	Ranker	0.874	7	ROM-KF, ROM-HF_KF_, CS-NF, ISOK-PT-ECC-KF_180_, ISOM-PT-Hip-Abd and CS-ML, CS-WF
Classifier subset evaluator (Naïve Bayes)	Best First	0.774	10	ISOK-PT-CON-KF_60_, Stature, ISOK-PT-CON-KE_180_, ISOK-PT-ECC-KF_60_, ISOK-PTECC-KF_180_, ISOK-PTECC-KE_60_, ISOM-PT-Hip-Abd, CS-ML, ROM-HIR, ROM-HER, ROM-HE, ROM-KF, ROM-AKDF_KE_ and ROM-AKDF_KF_
Consistency subset evaluator	Best First	0.699	5	ROM-HIR, ROM-HER, ROM-HE, ROM-KF and ROM-AKDF_KF_
Correlation attribute evaluator	Ranker	0.899	6	ROM-KF, ROM-HF_KF_, CS-ML, Stature, CS-NF and CS-CD
CV Attribute evaluator	Ranker	0.697	7	CS-ML, Dominant-leg, ISOK-PTECC-KF_60_, ROM-AKDF_KF_, ISOK-PTECC-KF_180_, ISOK-PTCON-KE_240_ and ISOK-PTECC-KE_30_
Gain ratio attribute evaluator	Ranker	0.865	6	CS-ML, ROM-KF, ROM-HF_KF_, Stature, ROM-HE and CS-CD
Info gain attribute evaluator	Ranker	0.874	6	ROM-KF, CS-ML, ROM-HF_KF_, ROM-HE, CS-CD and ISOK-PTECC-KF_180_
One R attribute evaluator	Ranker	0.857	7	ROM-KF, ROM-HF_KF_, CS-NF, ISOK-PTECC-KF_180_, CS-ML, ISOM-PT-Hip-Abd, CS-WF
Wrapper subset evaluator (Naïve Bayes)	Best First	0.851	9	Stature, ISOM-PT-Hip-Abd, CS-NF, CS-ML, CS-AP, ROM-HF_KF_, ROM-HER, ROM-HE, ROM-KF

BN: Bayesian Network Classifiers; AUC: area under the receiver operating characteristic curve; ISOK: isokinetic; KE: knee extensors; CON: concentric; ECC: eccentric; ISOM: isometric; PT: peak torque; Abd: abduction; ROM: range of motion; HF_KF_: hip flexion with the knee flexed; HE: Hip extension; HIR: hip internal rotation; HER: hip external rotation; KF: knee flexors; AKDF_KE_: ankle dorsi-flexion with the knee extended; AKDF_KF_: ankle dorsi-flexion with the knee flexed; CS: core stability; NF: unstable sitting without feedback; WF: unstable sitting with feedback; ML: unstable sitting while performing medial-lateral displacements with feedback; AP: unstable sitting while performing anterior-posterior displacements with feedback; CD: unstable sitting while performing circular displacements with feedback.

**Table 2:** Comparisons among the accuracy scores obtained by all the BN-based feature selection methods for the non-dominant leg. In grey is highlighted the best performing BN.

Feature selection algorithm	Search technique	AUC	N° of features selected	Description in ascending (from more to less important/relevant) order
-	-	0.821	31	S1 Table
Correlation-based feature subset evaluator	Best First	0.817	8	Dominant-leg, ISOM-Hip-Abd, CS-WF, CS-ML, ROM-HE, ROM-KF, ROM-AKDF_KE_ and ROM-AKDF_KF_
Chi squared attribute evaluator	Ranker	0.879	10	ROM-AKDF_KE_, ROM-AKDF_KF_, ROM-KF, ROM-HE, CS-ML, CS-CD, CS-WF, ROM-HF_KF_, ISOK-ECC-KF_180_ and CS-NF
Classifier attribute evaluator (Naïve Bayes)	Ranker	0.809	10	ROM-AKDF_KF_, ROM-KF, ROM-HE, ISOK-ECC-KF180, ROM-AKDF_KE_, ROM-HF_KF_, CS-WF, ISOK-ECC-KE_30_, ISOK-ECC-KE_60_ and CS-CD
Classifier subset evaluator (Naïve Bayes)	Best First	0.758	10	ISOK-ECC-KF_180_, ISOK-ECC-KE_60_, ISOM-Hip-Add, CS-NF, CS-WF, CS-CD, ROM-HE, ROM-KF, ROM-AKDF_KE_ and ROM-AKDF_KF_
Consistency subset evaluator	Best First	0.828	5	ROM-HABD, ROM-HIR, ROM-HER, ROM-KF and ROM-AKDF_KF_
Correlation attribute evaluator	Ranker	0.853	9	ROM-AKDF_KE_, ROM-AKDF_KF_, ROM-KF, CS-ML, ROM-HF_KF_, CS-WF, CS-NF, ISOM-Hip-Add and Dominant-leg
CV Attribute evaluator	Ranker	0.700	9	ROM-AKDF_KE_, Dominant-leg, ISOK-ECC-KF_180_, ISOK-ECC-_KF60_, ISOK-ECC-KE_30_, ISOK-CON-KE_240_, ISOK-ECC-KE_60_, ISOK-ECC-KF_30_ and ROM-AKDF_KF_
Gain ratio attribute evaluator	Ranker	0.853	10	ROM-AKDF_KE_, ROM-AKDF_KF_, ROM-KF, CS-ML, ROM-HF_KF_, CS-WF, CS-NF, Dominant-leg, ISOM-Hip-Add and ROM-HE
Info gain attribute evaluator	Ranker	0.853	9	ROM-AKDF_KE_, ROM-AKDF_KF_, ROM-KF, CS-ML, ROM-HE, CS-CD, ROM-HF_KF_, CS-WF, ISOK-ECC-KF_180_ and CS-NF
One R attribute evaluator	Ranker	0.731	9	ROM-AKDF_KF_, ROM-KF, ROM-HE, ISOK-ECC-KF_180_, ROM-AKDF_KE_, ISOK-ECC-KE_60_, ISOK-ECC-KF_60_, ISOK-CON-KF_240_ and ISOK-CON-KF_180_
Wrapper subset evaluator (Naïve Bayes)	Best First	0.809	22	ISOK-CON-KF_60_, Body-mass, ISOK-CON-KE_180_, ISOK-CON-KE_240_, ISOK-ECC-KF_30_, ISOK-ECC-KF_60_, ISOK-ECC-KF_180_, ISOK-ECC-KE_30_, ISOK-ECC-KE_60_, ISOM-Hip-Abd, ISOM-Hip-Add, CS-NF, CS-ML, CS-AP, CS-CD, ROM-HF_KF_, ROM-HF_KE_, ROM-HABD, ROM-HE, ROM-KF, ROM-AKDF_KE_ and ROM-AKDF_KF_

BN: Bayesian Network Classifiers; AUC: area under the receiver operating characteristic curve; ISOK: isokinetic; KE: knee extensors; CON: concentric; ECC: eccentric; ISOM: isometric; PT: peak torque; Abd: abduction; ROM: range of motion; HF_KF_: hip flexion with the knee flexed; HE: Hip extension; HIR: hip internal rotation; HER: hip external rotation; KF: knee flexors; AKDF_KE_: ankle dorsi-flexion with the knee extended; AKDF_KF_: ankle dorsi-flexion with the knee flexed; CS: core stability; NF: unstable sitting without feedback; WF: unstable sitting with feedback; ML: unstable sitting while performing medial-lateral displacements with feedback; AP: unstable sitting while performing anterior-posterior displacements with feedback; CD: unstable sitting while performing circular displacements with feedback.

**Fig 1:**
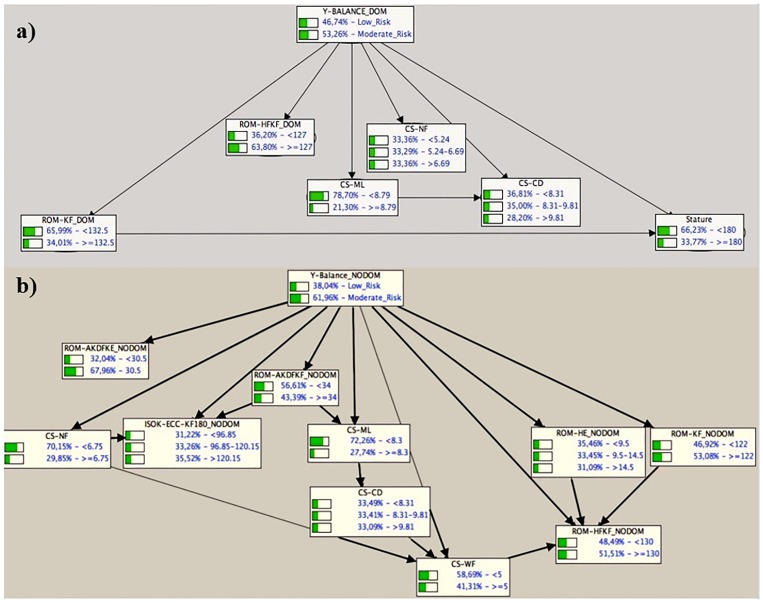
Directed acyclic graphs corresponding to the dynamic postural control BNs built for the dominant leg (Fig 1a) and non-dominant leg (Fig 1b). The a priori probability distributions for each feature are given, where the likelihood for each feature’s label is expressed in percentage.

Fig 1 presents the directed acyclic graphs (DAGs) corresponding to the dynamic postural control BNs built for the dominant (Fig 1a) and non-dominant leg (Fig 1b). In addition, both DAGs also show the a priori probability distributions (expressed in percentages), that is, without entering any observed value, for each of the two or three labels of the six and ten variables selected to build the dynamic postural control BNs. Thus, for the class variable of the dominant leg (Y-BALANCE_DOM), six child nodes or independent predictors were observed: knee flexion (ROM-KF_DOM) and hip flexion with knee flexed (ROM-HF_KF__DOM) ROMs, core stability measures recorded while performing medial-lateral (CS-ML) and circular (CS-CD) displacements with feedback, and also without displacement and nor feedback (CS-NF), and stature. Likewise, what can also be observed is the presence of connections between hip flexion ROM and the players´ stature (ROM-KF_DOM → Stature) as well as between the measures of core stability assessed while performing medial-lateral (CS-ML) and circular (CS-CD) displacements (CS-ML → CS-CD). The DAG corresponding to the dynamic postural control BN of the non-dominant leg shows the presence of nine child nodes, corresponding to five ROM (ankle dorsiflexion with knee extended [ROM-AKDF_KE__NODOM] and flexed [ROM-AKDF_KE__NODOM], knee flexion [ROM-KF_NODOM] and hip extension [ROM-HE_NODOM] and flexion with knee flexed [ROM-HF_KF__NODOM] ROMs), three core stability measured during both static (unstable sitting with [CS-WF] and without [CS-NF] feedback) and dynamic tasks (unstable sitting while performing medial-lateral displacements with feedback [CS-ML]) and one isokinetic strength (eccentric knee flexors peak torque [ISOK-ECC-KF_180__NODOM]) measures. Likewise, a number of connections among variables were also displayed in the DAG for the dynamic postural control BN of the non-dominant leg (e.g.: CS-NF → ISOK-ECC-KF_180__NODOM, ROM-KF_NODOM → ROM-HF_KF__NODOM). Another child node was observed, the measure of core stability assessed while performing circular displacements with feedback (CS-CD), that acts as descendent of another measure of core stability, in its case the one measured while performing medial-lateral displacements (CS-ML).

The individual contribution of each label of the different variables finally selected on the probability of having the class variable (y-balance test composite score) in its low and moderate risk states is shown in Table 3 for both the dominant and non-dominant legs. Knee flexion ROM (≥132.5°) and core stability assessed while performing medial-lateral displacements with feedback (≥8.79 mm) measures were the ones that presented the highest impact on the probability of having the class variable of the dominant leg in its low (84.34%) and moderate risk (95.01%) states, respectively. Hip extension (≥14.5°) and ankle dorsiflexion with knee extended (<30.5°) ROM measures were also the predictors with the highest contribution to have the class variable of the non-dominant leg in its low (62.84%) and moderate risk (96.7%) states, respectively.

**Table 3:** Individual contribution of each level of the final variables selected on the probability of having the class variable (y-balance composite score) of the non-dominant leg in its low and moderate risk states. In grey are highlighted the labels of the variables that present the highest individual contribution of having the class variable in its low and moderate risk scores.

	Y-balance (composite score)	
	Low risk	Moderate risk
	**Dominant leg**	
No instantiations	46.74	53.26
ROM-KF (°)		
▪ <132.5	27.36	72.64
▪ ≥132.5	84.34	15.66
ROM-HF_KF_ (°)		
▪ <127	14.67	85.33
▪ ≥127	64.94	35.06
CS-ML (CoP mm)		
▪ <8.79	58.04	41.96
▪ ≥8.79	4.99	95.01
Stature (cm)		
▪ <180	56.55	43.45
▪ ≥180	23.27	76.73
CS-NF (CoP mm)		
▪ <5.24	34.25	65.75
▪ 5.24–6.09	71.76	28.24
▪ ≥6.09	34.25	65.75
CS-CD (CoP mm)		
▪ <8.31	52.04	47.96
▪ 8.31–9.81	62.74	37.26
▪ ≥9.81	18.92	81.8
	**Non-dominant leg**	
No instantiations	38.04	61.96
ROM-AKDF_KE_ (°)		
▪ <30.5	3.3	96.7
▪ ≥30.5	54.42	45.58
ROM-AKDF_KF_ (°)		
▪ <34	18.23	81.77
▪ ≥34	61.79	38.21
ROM-KF (°)		
▪ <122	15.77	84.23
▪ ≥122	57.74	42.26
ROM-HE (°)		
▪ <9.5	31.8	68.11
▪ 9.5–14.5	21.52	78.48
▪ ≥14.5	62.84	37.16
CS-ML (CoP mm)		
▪ <8.3	48.26	51.74
▪ ≥8.3	11.43	88.57
CS-CD (CoP mm)		
▪ <8.31	47.13	52.87
▪ 8.31–9.81	42.6	57.4
▪ ≥9.81	24.25	75.75
CS-WF (CoP mm)		
▪ <5	47.97	52.03
▪ ≥5	25.56	74.44
ROM-HF_KF_ (°)		
▪ <130	25.61	74.39
▪ ≥130	52.26	47.74
ISOK-ECC-KF_180_ (Nm)		
▪ <96.85	20.95	79.05
▪ 96.85–120.15	56.45	43.55
▪ ≥120.15	35.45	64.55
CS-NF (CoP mm)		
▪ <6.75	46.7	53.3
▪ ≥6.75	17.7	82.3

ISOK: isokinetic; KE: knee extensors; ECC: eccentric; ROM: range of motion; HF_KF_: hip flexion with the knee flexed; HE: Hip extension; KF: knee flexors; AKDF_KE_: ankle dorsi-flexion with the knee extended; AKDF_KF_: ankle dorsi-flexion with the knee flexed; CS: core stability; NF: unstable sitting without feedback; WF: unstable sitting with feedback; ML: unstable sitting while performing medial-lateral displacements with feedback; CD: unstable sitting while performing circular displacements with feedback.

In Table 4 it can be seen that by mean of a belief updating process which uses two different configurations (i.e.: the process by which new evidence is introduced in some target variables of the model), it was possible to achieve the maximal hypothetical probability (98.98%) that a futsal player will show a limited (moderate risk) dynamic postural control performance of the dominant leg, which implies a “jump” of approximately 45 percentage points from the initial value shown within the studied population. Table 4 also displays how through three instantiations it is possible to achieve the maximal hypothetical probability that a player would have a dynamic postural control performance of the dominant leg that might be categorized as “low risk for lower-extremity injuries” (98.08%), with an increase of approximately 52 percentage points from the initial value. Similarly, Table 5 presents another step-by-step belief updating process carried out to maximize both labels (low risk and moderate risk) of the class variable for the dynamic postural control model of the non-dominant leg. In particular, only two variables need to be observed (fixed) to achieve the greatest hypothetical probability (99.29%) that a player would have a limited dynamic postural control performance (moderate risk). However, the correct value must be entered for 5 variables to maximize the probability (98.65%) that a player would have a dynamic postural stability performance categorized as “low risk for lower-extremity injuries”, which suppose an increase of approximately 60 percentage points with respect to its initial probability (38.04%). For the belief updating process carried out in both BNs and shown in Tables 4 and 5, an intercausal reasoning (when different causes of the same effect can interact) was applied. From each step, the variable and the state that induces the greatest increase in the likelihood of the class variable to show a low and moderate state were chosen.

**Table 4:** Step-by-step instantiations leading to maximization of the likelihood of having the class variable (y-balance) of the dominant leg in its low and moderate risk categories.

Step	Instantiate variable	Label	y-balance
			**Moderate risk**
1	None		53.26%
2	CS-ML	≥8.79	95.01%
3	ROM-HF_KF__DOM	<127	98.98%
			**Low risk**
1	None		46.74%
2	ROM-KF_DOM	≥132.5	84.34%
3	ROM-HF_KF__DOM	≥127	91.91%
4	CS-NF	5.24–6.69	97.05%

CS: core stability; ML: unstable sitting while performing medial-lateral displacements with feedback; ROM: range of motion; HF_KF_: hip flexion with the knee flexed; KF: knee flexors; DOM: dominant leg; NF: no feedback.

**Table 5:** Step-by-step instantiations leading to maximization of the likelihood of having the criterion variable (y-balance) of the non-dominant leg in its low and moderate risk states.

Step	Instantiate variable	Label	y-balance
			**Moderate risk**
1	None		61.96%
2	ROM-AKDF_KE__NONDOM	<30.5	96.7%
3	CS-ML	≥8.3	99.29%
			**Low risk**
1	None		38.04%
2	ROM-HE_NODOM	>14.5	63.84%
3	ISOK-ECC-KF_180__NODOM	96.85–120.15	81.54%
4	ROM-AKDF_KF__NONDOM	≥34	94.32%
5	ROM-AKDF_KE__NONDOM	≥30.5	97.03%
6	ROM-KF_NONDOM	≥122	98.65%

CS: core stability; ML: unstable sitting while performing medial-lateral displacements with feedback; ROM: range of motion; KF: knee flexors; AKDF_KE_: ankle dorsi-flexion with the knee extended; AKDF_KF_: ankle dorsi-flexion with the knee flexed; HE: hip extension; ISOK: isokinetic strength; ECC: eccentric; NONDOM: non-dominant leg.

**Fig 2:**
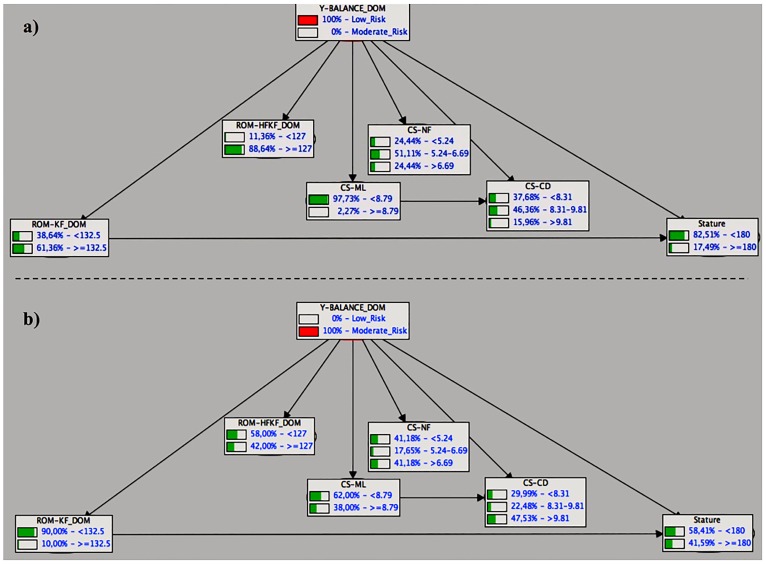
A top-down reasoning for the dynamic postural control BNs of the dominant leg in which the class variable (y-balance composite scores) was instantiated in their two labels: a) low risk and b) moderate risk.

**Fig 3:**
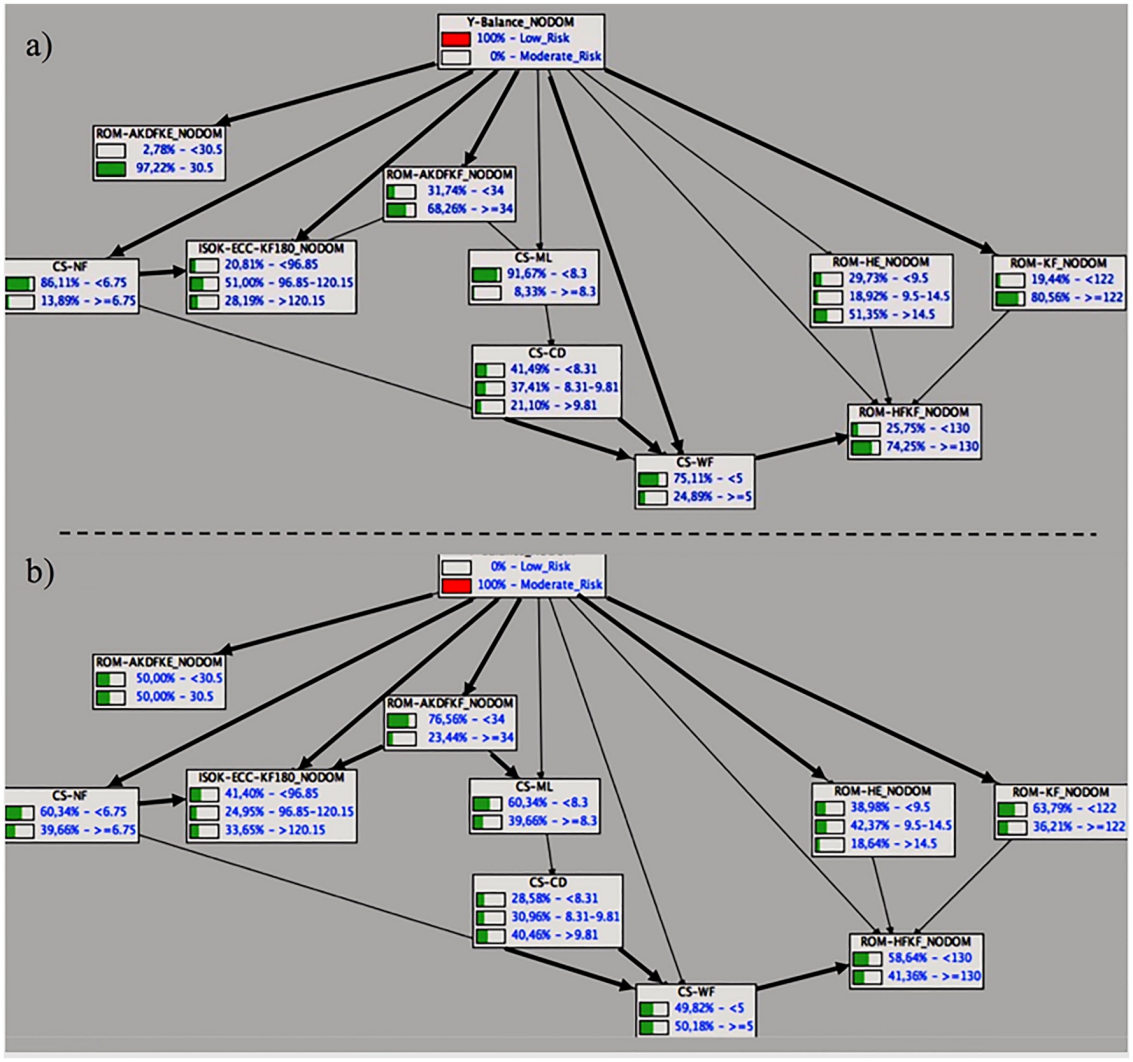
A top-down reasoning for the dynamic postural control BNs of the non-dominant leg in which the class variable (y-balance composite scores) was instantiated in their two labels: a) low risk and b) moderate risk.

Finally, Fig 2 (dominant leg) and 3 (non-dominant leg) show a top-down reasoning for the dynamic postural control BNs in which in both cases, the class variable (y-balance composite scores) was instantiated in their two labels in order to define / predict a profile. For the dynamic postural control BN of the dominant leg, Fig 2 shows that when the class variable is instantiated at is maximum of “low risk” (Fig 2a), three variables or father nodes show a clearly imbalanced distribution of probabilities in favor of one of their labels (ROM-HF_KF__DOM, CS-ML and stature). In particular, a futsal player with a dynamic postural control performance categorized as “low risk” is very likely to have a hip flexion with knee flexed ROM higher than 127°, a core stability score (measured while performing medial-lateral displacements) lower than 8.79 mm (mean radial error) and a stature shorter than 180 cm. Subsequently, Fig 2b also shows that when the label “high risk” of the class variable is instantiated, only knee flexion ROM reported a clear imbalance in the distribution of probabilities between its two labels (in favour to the label “<132.5°”) and hence, a high-risk profile was not visually clear. Regarding the dynamic postural control BN of the non-dominant leg, Fig 3 shows that when the class variable is instantiated in its “low risk” label (Fig 3a), seven out of nine variables present a clearly imbalanced distribution orientated to one of their labels. Thus, there seems to be a low risk profile characterised by moderate to high ROM values for the ankle, knee and hip (flexion) joints alongside with a high core stability performance during static and dynamic tasks. Contrarily, when the moderate risk label was instantiated (Fig 3b), it was not possible to find a clear profile.

## Discussion

The BNs generated using the selected features by the algorithms correlation attribute evaluator and chi squared reported the highest evaluation criteria for the dominant (AUC = 0.899) and non-dominant (AUC = 0.879) legs, respectively. The ability of both BNs to classify the instances correctly into one of the two categories of the class variable (low risk vs. moderate risk) cannot be compared with the models developed (through regression logistic techniques) in previous studies because neither of them reported any measure of their global ability or accuracy.

The BN built for the dynamic postural control of the dominant leg identified six independent predictors: knee flexion and hip flexion with the knee flexed ROMs, stature, and one static (with feedback) and two dynamic (assessed while performing medial-lateral and circular displacements with feedback) core stability measures. On the contrary, the feature selection-based BN of the dynamic postural control of the non-dominant leg shows nine father nodes or independent predictors for the distance reached in the y-balance test: five of them were ROMs (hip flexion and extension with knee flexed, knee flexion and ankle dorsiflexion with knee flexed and extended), three were static (with and without feedback) and dynamic (assessed while performing medial-lateral displacements) core stability measures and one was a measure of the isokinetic eccentric strength of the knee flexors. Therefore, the performance achieved in the y-balance test (independent of the leg) and consequently, the dynamic postural control, appears to be widely influenced by the hip and knee flexion and the ankle dorsiflexion ROM measures, all in the sagittal plane, as well as by measures of static but mainly dynamic core stability in the frontal plane. In particular, the highest label of the dynamic core stability measure (the higher the value the worse the core stability) recorded while performing medial-lateral displacements (≥8.9 mm) and the lowest label of the hip flexion with knee flexed ROM (<127°) were the two neuromuscular parameters that presented the largest individual contribution (an increase of 41.7 and 32.1 percentage points, respectively) to the probability that the class variable of the dominant leg (y-balance composite score) would adopt its moderate risk category. For the non-dominant leg, the two measures that have the highest impact on the probability of having the class variable in its moderate risk category were the lowest label of the ankle dorsiflexion with knee extended ROM (<30.5°) and again, the highest label of the dynamic core stability measure recorded while performing medial-lateral displacements (≥8.3 mm).

These results are in agreement with the findings reported by previous studies [17,21,46] which found that the hip and knee flexion and ankle dorsiflexion ROMs individually determined a meaningful proportion of the explained variance (*R*^*2*^) for the y-balance test (ranging from 5 to 30% of the composite score) in different cohorts of athletes. These findings may support the hypothesis that those athletes with limited hip and knee flexion and ankle dorsiflexion ROMs might show a sub-optimal dynamic postural control while performing explosive actions (i.e., kicking and changes of direction) due to a smaller anterior displacement of their center of mass, which may increase the likelihood of losing stability.

Although core stability has been proposed as a crucial factor for y-balance test [47], only López-Valenciano et al. [17] have confirmed this link in professional female football players. In particular, this study found that the measure of core stability recorded while players were performing medial-lateral displacements on an unstable seat explained a large percentage (31.1%) of the performance achieved in the composite score of the y-balance test in female, but not in male professional football players. These sex-related differences found by López-Valenciano et al. [17] in the identification of this variable as an independent predictor for the y-balance test performance, but not in the absolute distances reached (composite scores), may be partially attributed to the fact that female players reported better results (statistically significant) in the core stability measures (with the exception of the static stability measure with feedback [CS-WF]) in comparison with their counterpart male football players (e.g.: CS-NF: 6.1 mm [males]– 4.3 mm [females], CS-CD: 10.8 mm [males]– 9.2 mm [females]). These differences in the core stability results in favor of the female players might have allowed them to develop different neuromuscular strategies to control the trunk in the frontal plane more efficiently while performing functional unilateral movements (e.g. changes of direction, kicking). Consequently, the individual contribution of the different measures of neuromuscular performance on dynamic postural control might have been modified, so core stability may have now adopted a more relevant role in such cohort of female players in contrast to other parameters (e.g. ROM). This hypothesis seems to be supported by the results reported in the current study, in which the scores obtained by the male futsal players in the core stability tasks were similar or even slightly better to those reported by López-Valenciano et al. [17] for the female players, and both BNs also selected some of these measures as independent predictors for the dynamic postural control performance.

Thanks to the fact that BNs have the ability to make simulations or instantiations when new evidence is introduced in the model, it was possible to carry out the study of the simplest step-by step combination of instanced variables (in term of the number of instantiations made) to maximize the probability for the class variable (composite score) to have its low and moderate category for the dominant (Table 4) and non-dominant legs (Table 5). The combination of poor dynamic core stability scores (medial-lateral displacement) (≥8.79 and 8.3 mm for the dominant and non-dominant leg, respectively) with limited hip flexion with knee flexed (dominant leg) (<127°) or ankle dorsiflexion with knee flexed (non-dominant leg) (<30.5°) ROM measures presented a strong probabilistic and negative relationship with dynamic postural control. On the contrary, the combination of high hip (>127°) and knee (>132.5 and 122° for the dominant and non-dominant leg, respectively) flexion and ankle dorsiflexion with knee flexed (>34°) and extended (>30.5°) ROM values seems to have presented the strongest probabilistic and positive impact on dynamic postural control.

### Limitations

The current findings are limited to the participants’ sport background (elite futsal players) so the extrapolation to other sport cohorts should be made with a certain degree of caution. Each sport modality and level of competition requires differences in technical skills, specific movements, training load and physical capacities, all of which predispose athletes to individual chronic musculo-skeletal adaptations, thus possibly developing different strategies for neuromuscular control and influencing subsequent y-balance test scores.

## Conclusion

The BNs built (AUC = 0.899 and 0.879 for the dominant and non-dominant legs respectively) in the current study demonstrated that the dynamic postural control in elite male futsal players presents a strong relationship to the abilities to flex the hip, knee and ankle (dorsiflexion) joints in the sagittal plane and to control the core structures during static, but mainly during dynamic actions in the frontal plane. Therefore, training interventions aimed at improving or maintaining unilateral dynamic balance in professional male football players should include, among other things, exercises (i.e. stretching exercises for the major muscles of the posterior chain) that allow futsal players to achieve hip and knee flexion and ankle dorsiflexion with knee flexed and extended ROM scores equal or higher than 127°, 132.5°, 34° and 30.5°, respectively. Likewise, these training interventions should also include exercises to maintain or improve both the static (e.g. frontal, back and side planks) and dynamic medial-lateral (e.g. plank jacks and Russian twists, one-legged squats, lunges, airplane exercises) core stability so that futsal players can achieve medial radial error values lower than 6.69 and 8.79 mm, respectively.

## Supporting information

S1 Table Description of the features recorded to build the Bayesian Networks. (DOCX)

S1 File Database dominant leg. (ARFF)

S2 File Database non-dominant leg. (ARFF)
