# Supplementary material for: A Bayesian Network approach to study the relationships between several neuromuscular performance measures and dynamic postural control in futsal players
Source: PLoS One. 2019 Jul 25;14(7):e0220065. doi: 10.1371/journal.pone.0220065 (PMC6657865; doi:10.1371/journal.pone.0220065)
Supplement: S1 Table — (DOCX) [file pone.0220065.s001.docx]

**S1: Description of the features recorded to build the Bayesian Networks.**

| **Name** | **Labels** | | |
| --- | --- | --- | --- |
|  | **Dominant leg** | **Non-dominant leg** |  |
| **Y-balance (composite score)** | High risk (<89.6%) or Low risk (≥89.6%) | | |
| **Personal characteristics:** |  |  |  |
| 1. Dominant leg | Left or right | | |
| 1. Stature (cm) | <180 or ≥180* | <173.55, 173.55-179.35 or >179.35 |  |
| 1. Body mass (kg) | <70.1, 70.1-74.95 or >74.95 | <70.1, 70.1-74.95 or >74.95 |  |
| **Isometric hip abduction and adduction strength (N/kg):** | |  |  |
| 1. ISOM-Hip-Abd | <2.73, 2.73-2.93 or >2.93 | <2.55, 2.55-2.81 or >2.81 |  |
| 1. ISOM-Hip-Abd | <2.61, 2.61-3.27 or >3.27 | <3 or ≥3* |  |
| **Lower extremity ranges of motion (º):** | |  |  |
| 1. ROM-HF_KF_ | <127 or ≥127* | <130 or ≥130* |  |
| 1. ROM-HF_KE_ | <70.5, 70.5-79.5 or >79.5 | <70.5, 70.5-81 or >81 |  |
| 1. ROM-HAB | <56, 56-63.5 or >63.5 | <51.5, 51.5-60.5 or >60.5 |  |
| 1. ROM-HIR | <39.5, 39.5-44.5 or >44.5 | <34.5, 34.5-44.5 or >44.5 |  |
| 1. ROM-HER | <51.5, 51.5-59.5 or >59.5 | <49.5, 49.5-58 or >58 |  |
| 1. ROM-HE | <9, 9-14 or >14* | <9.5, 9.5-14.5 or >14.5 |  |
| 1. ROM-KF | <132.5 or ≥132.5* | <122 or ≥122* |  |
| 1. ROM-AKDF_KE_ | <31 or ≥31* | <30.5 or ≥30.5* |  |
| 1. ROM-AKDF_KF_ | <32.5, 32.5-37.5 or >37.5 | <34 or ≥34* |  |
| **Core stability (mm):** |  |  |  |
| 1. CS-NF | <5.24, 5.24-6.69 or >6.69 | <6.75 or ≥6.75* |  |
| 1. CS-WF | <3.66, 3.66-5.34 or >5.34 | <5 or ≥5* |  |
| 1. CS-ML | <8.79 or ≥8.79* | <8.3 or ≥8.3 |  |
| 1. CS-AP | <6.88, 6.88-7.96 or >7.96 | <6.88, 6.88-7.96 or >7.96 |  |
| 1. CS-CD | <8.31, 8.31-9.81 or >9.81 | <8.31, 8.31-9.81 or >9.81 |  |
| **Isokinetic knee flexion and extension strength (Nm):** | |  |  |
| 1. ISOK-CON-KF_60_ | <98.95, 98.95-113.95 or >113.95 | <92.45, 92.45-112 or >112 |  |
| 1. ISOK-CON-KF_180_ | <84.2, 84.2-106.05 or >106.05 | <80.8, 80.8-106.65 or >106.65 |  |
| 1. ISOK-CON-KF_240_ | <82.65, 82.65-104 or >104 | <80.35, 80.35-100.35 or >100.35 |  |
| 1. ISOK-CON-KE_60_ | <172.6, 172.6-220 or >220 | <175.4, 175.4-204.15 or >204.15 |  |
| 1. ISOK-CON-KE_180_ | <124.85, 124.85-149.5 or >149.5 | <127, 127-145.4 or >145.4 |  |
| 1. ISOK-CON-KE_240_ | <112, 112-142.65 or >142.65 | <116.55, 116.55-134.05 >134.05 |  |
| 1. ISOK-ECC-KF_30_ | <98, 98-130.25 or >130.25 | <97.45, 97.45-119.65 or >119.65 |  |
| 1. ISOK-ECC-KF_60_ | <79.95, 79.95-102.4 or >102.4 | <101.3, 101.3-126.05 or >126.05 |  |
| 1. ISOK-ECC-KF_180_ | <103.4, 103.4-124.45 or >124.45 | <96.85, 96.85-120.15 or >120.15 |  |
| 1. ISOK-ECC-KE_30_ | <218.9, 218.9-268.75 or >268.75 | <222.45, 222.45-268.35 or >268.35 |  |
| 1. ISOK-ECC-KE_60_ | <217.75, 217.75-262.95 or >262.95 | <223, 223-267.2 or >267.2 |  |
| 1. ISOK-ECC-KE_180_ | <191.75, 191.75-246.25 or >246.25 | <188.95, 188.95-238.45 or >238.45 |  |

*: discretization based on visual inspection; N: Newton; m: meter, º: degrees; cm: centimeter; kg: kilograms; ISOM: isometric; PT: peak torque; Abd: abduction; Add: adduction; ROM: range of motion; HFKF: hip flexion with the knee flexed; HFKE: hip flexion with the knee extended; HE: Hip extension; HABD: hip abduction at 90º of hip flexion; HIR: hip internal rotation; HER: hip external rotation; KF: knee flexors; AKDFKE: ankle dorsi-flexion with the knee extended; AKDFKF: ankle dorsi-flexion with the knee flexed; CS: core stability; NF: unstable sitting without feedback; WF: unstable sitting with feedback; ML: unstable sitting while performing medial-lateral displacements with feedback; AP: unstable sitting while performing anterior-posterior displacements with feedback; CD: unstable sitting while performing circular displacements with feedback; ISOK: isokinetic; KE: knee extensors; CON: concentric; ECC: eccentric.
